# Emerging Animal-Associated Fungal Diseases

**DOI:** 10.3390/jof8060611

**Published:** 2022-06-08

**Authors:** Julia Eva Carpouron, Sybren de Hoog, Eleni Gentekaki, Kevin David Hyde

**Affiliations:** 1 Center of Excellence in Fungal Research, Mae Fah Luang University, Chiang Rai 57100, Thailand; 6271105502@lamduan.mfu.ac.th (J.E.C.); gentekaki.ele@mfu.ac.th (E.G.); 2 School of Science, Mae Fah Luang University, Chiang Rai 57100, Thailand; 3 Centre of Expertise in Mycology, Radboud University Medical Centre/Canisius Wilhelmina Hospital, 6525 GA Nijmegen, The Netherlands; sybren.dehoog@radboudumc.nl; 4 Institute of Plant Health, Zhongkai University of Agriculture and Engineering, Haizhu District, Guangzhou 510225, China; 5 Mushroom Research Foundation, 128 M.3 Ban Pa Deng T. Pa Pae, A. Mae Taeng, Chiang Mai 50150, Thailand

**Keywords:** domesticated animals, environmental pathogens, outbreaks, wildlife, zoonoses

## Abstract

The Global Action Fund for Fungal Infections (GAFFI) estimates that fungal diseases kill around 150 people each hour, and yet they are globally overlooked and neglected. *Histoplasma* and *Talaromyces*, which are associated with wildlife, cause systemic infections that are often lethal in patients with impaired cellular immunity. Dermatophytes that cause outbreaks in human hosts are often associated with domesticated animals. Changes in human behavior have been identified as a main cause of the emergence of animal-associated fungal diseases in humans, sometimes caused by the disturbance of natural habitats. An understanding of ecology and the transmission modes of causative agents is therefore essential. Here, we focus on fungal diseases contracted from wildlife and domesticated animals, their habitats, feces and carcasses. We discuss some basic fungal lifestyles and the risk of transmission to humans and illustrate these with examples from emerging and established diseases.

## 1. Introduction

Fungal diseases affect around one billion people annually resulting in over 1.5 million deaths [1,2]. Since the mid-20th century, a global rise in fungal infections and associated deaths has been noted. In recent years, several new and known fungal infections have emerged in immunocompromised and other long-term patients and in the human population as a whole [3,4]. Although some intrinsic characteristics of fungi contribute to the emergence of diseases [5], recent scenarios have mainly been associated with our modern activities, including the widespread use of medication (e.g., corticosteroids), urbanization, domestication, increase in population, tourism, migration and accelerated climate change [6,7,8,9,10]. Several of these emergent fungal infections have been described as zoonoses. Animals have been construed as the main contributors to infectious human diseases, representing 75% of all emerging infections [11]. Nonetheless, this fraction does not separate true animal pathogens from environmental pathogens that thrive on fecal matter and decaying animal bodies. This is a considerable oversight, as many emergent infectious fungi have an environmental reservoir with the capacity to invade animal hosts, including humans [3,12,13]. For instance, *Talaromyces marneffei*, an ascomycete infecting over 50,000 HIV-positive patients yearly, has been linked to bamboo rats and their habitats [14,15,16,17,18]. However, animals should not be falsely accused, our modern activities may have more impact, encroaching into wild animals’ habitats and domesticating them as pets represents a larger threat to public health [19]. In this context, we provide an update on the terminology and trends of fungal diseases emerging in humans, related to animals. For each category of fungus, we examine and compare the mode and likelihood of transmission to humans.

## 2. Terminology for Animal-Associated Fungal Infections

**Figure 1:**
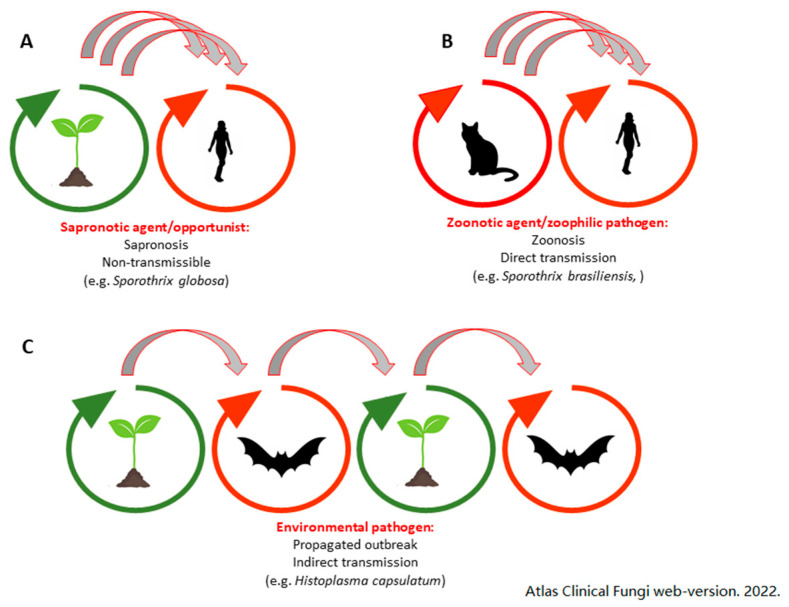
Lifecycle and mode of transmission of zoonotic agent, sapronotic agent and environmental pathogen. (**A**) sapronotic agents/opportunists are non-transmissible; outbreak occurs following repeated infection from a common environmental source leading to sapronosis; (**B**) zoonotic agents/zoophilic pathogens depend on the host for feeding and transmission, which mainly occurs directly via contagious animal hosts; (**C**) environmental pathogen, feeding and sexuality is environmental, propagation via host, non-contagious. In (**A**,**B**), repeated events of transmission occur from the same host/environment as opposed to (**C**), where a single host is infected and spreads the infective agent in an environment; this is represented by the number of arrows connecting each host and environment. Adapted with permission from ref. [29]. Copyright 2022 Atlas Clinical Fungi web-version.

Several terms are used to refer to infectious diseases associated with animals. “Zoonosis” and “sapronosis” have often been used with variable and overlapping meanings by different authors [20,21]. The World Health Organization (WHO) officially describes zoonoses as infections naturally transmitted between vertebrate animals and humans. The term thus does not distinguish whether “true” pathogens or opportunists are involved [21,22,23]. Pathogens are adapted to and can complete their life cycle in a mammalian host, whereas opportunists inhabit environmental niches but are also able to survive in animal hosts. When an opportunist, acquired from a non-living source such as soil, decaying plant material or feces, causes infection and/or an outbreak, the latter is referred to as a sapronosis [22]. None of the fungi are dependent on vertebrate hosts, but they have increased fitness if they use a mammal in any stage of their life cycle. Sapronotic agents occasionally infect humans or other animals without showing any specialized adaptation to their host [24,25,26]. Fungi that have double life cycles with stages in both the environment and an animal host are known as environmental pathogens. These live as saprobes, but once inside a warm-blooded vertebrate, they display a specialized invasive phase adapted to the host [22,27]. Following infection, environmental pathogens can be dispersed in the environment through defecation (e.g., *Histoplasma capsulatum*) [28] or potentially escape the host’s body upon death (e.g., *Coccidioides immitis*) [22]. On the other hand, opportunistic fungi are non-transmissible among hosts, and upon death of the host, the fungus dies as well [22]. Environmental pathogens, zoonotic agents and sapronotic agents differ significantly in their life cycle, target population and clinical symptoms, and a clear distinction between them is important [22,26] (Figure 1).

## 3. Direct versus Indirect Transmission—Should We Fear One More than the Other?

Among other factors, the emergence of an infectious disease depends on the opportunity of transmission, and thus the emergence is dependent on transmission mode [30]. Two modes of transmission are recognized in infectious diseases including zoonoses: direct and indirect. Skin contact, scratches and bites constitute examples of direct transmission from an infected animal to a human host [20]. It is a highly efficient and exclusive mode of transmission in the spread of *Sporothrix brasiliensis* by cats. Indirect transmission occurs when pathogens shed in the environment by an infectious host are acquired by a new host [31,32]. The latter type of transmission involves vehicles for dispersal, such as air, water, food or an animal vector [20]. For instance, bats are vectors of *Histoplasma capsulatum*, as they actively carry and spread the fungus during long-distance flights. When comparing the two modes of transmission, indirectly transmitted pathogens are generally more successful long-term spreaders. Indeed, indirect transmission often involves extensive environmental dissemination. This is particularly true for dimorphic fungi and fungi producing spores that can persist outside the host for extended periods, thus increasing the likelihood of transmission [30]. Persistence in the environment also increases the probability of spillover events resulting in infections of not only larger populations, but a wider host range as well [30]. Routes of transmission also dictate the prevalence and risk of outbreak in human populations. The prevalence of infection in the primary animal host population, the rate of encounter of these animals with humans and the probability of humans becoming infected upon contact constitute outbreak risks [33]. However, it should be noted that several fungi are not restricted to one mode of transmission [30]. For example, some dermatophytes are highly contagious and can easily jump to a new host by direct contact, but can also survive for extended periods on keratinized fomites to be transmitted indirectly [23,34]. The adaptation of zoophilic dermatophytes to the human host may occur within a rather short time span [35].

## 4. Are Fungal Pathogens of Close Human Relatives More Likely to Cause Outbreaks in Humans?

Pathogens often coevolve with their hosts; thus, many pathogenic microorganisms are adapted to and infect a limited number of host species [36,37]. Specific ligand–receptor interactions between an infectious agent and its host must occur for a pathogen to access and multiply on an infectable surface; colonize, invade and multiply inside the host; and resist its innate and/or adaptive immune mechanisms. Following this concept, for the successful invasion of a new category of hosts, the pathogen has to re-adapt to some or all of these interactions. For pathogens outside the fungal kingdom, it has been hypothesized that those of non-human primates have a higher probability to cross the species barrier and infect humans, as opposed to those associated with phylogenetically more distant hosts [38]. In support of this, in proportion to the number of species in the same taxon, pathogens of non-human primates contribute more to human infectious diseases than those of non-primate mammals [36,38]. This barrier to transmission of pathogens between species is referred to as the “species barrier”, indicating the host specificity of the agent. In fungi, this is known, e.g., in *Pneumocystis* [39], where the numerous extant species have restricted host ranges, and the human-associated species, *P. jirovecii*, has been found to infect humans only [40]. However, this concept of specificity and species barrier is less applicable to fungi, which seem to be less dependent on particular hosts. For instance, *Microsporum canis,* a pathogen of cats, dogs and horses is also transmissible between humans [37], causing small, self-limiting outbreaks in school children [41,42].

Janzen [43] introduced the concept of “ecological fitting”, which seems to provide a suitable alternative explanation for host shifts in fungi. In the context of host–pathogen interaction, “ecological fitting” is the mechanism whereby the pathogen persists in the novel non-optimal environment of the new host, using existing traits that have evolved elsewhere and in response to a different set of environmental conditions [44,45]. Ecological fitting may occur through two scenarios. First, through similarity in the resources provided by the previous and novel host, referred to as ecological fitting via resource tracking. From a pathogen’s perspective, the new host may actually not be so different from the previous one, as long as essential conditions for infection are the same [36,46]. Here, similarities can be closely linked to the phylogenetic relatedness of the hosts [47]. However, several pathogenic fungi also have the ability to survive and adapt under sub-optimal conditions, e.g., temperature [48]. In the second scenario, fungi have what is known as a broad “sloppy fitness space”, which facilitates new associations between species or simply expands the number of potential hosts: ecological fitting via sloppy fitness space [45]. The main limitation is that for the fungal pathogens to jump to humans, their primary host must also be a mammal, as host shifts from other vertebrates such as birds, amphibians and fish are highly exceptional. Most environmental fungal pathogens of rodents, bats or armadillos have a sufficiently wide fitness space to be able to infect humans [48].

## 5. Some Examples of Animal-Associated Fungal Infections

In recent years, the epidemiological patterns of some fungal diseases associated with domesticated and wild animals have changed, showing an increased prevalence, death toll or change in populations at risk. Here, we summarize and review new insights and findings of some important animal-associated fungal infections. Using specific examples, we delve into the concepts discussed above: mode of transmission, host shift and classification of causative fungal pathogen in relation to the risk of outbreaks and threat to public health.

### 5.1. Dermatophytoses

Dermatophytosis, commonly known as ringworm or tinea, occurs worldwide, but currently, a change is being observed in the epidemiology of the disease. The distribution of species causing the disease now varies significantly between countries. This has been attributed to inappropriate treatment, high population density, traveling and migration, among other factors [49,50,51]. With the domestication of animals as pets, new species such as *T. erinacei* and *T. benhamiae* have also emerged in humans, causing an increase in zoonotic infections [25,52]. Concerns arise as the elevated cost and lengthy treatment of dermatophytosis frequently leads to a non-adherence to therapy, with an emergence of resistance as a serious consequence [53].

Recently, considerable alarm has been raised due to an ongoing large-scale dermatophytosis outbreak in India, which is spreading to other countries [54]. The disease spreads among humans, typical of anthropophilic causative agents, but manifests a severity of symptoms typical of a zoophilic dermatophyte. The outbreak has been attributed to a novel species, *Trichophyton indotineae* [55], which shows increased virulence as opposed to the previously widespread *T. mentagrophytes* and *T. interdigitale* [54]. Tang et al. [35] have suggested that what is being observed is the geophilic, wild animal-infecting species, *T. mentagrophytes*, shifting host to domesticated animals and now behaving as an anthropophilic clonal offshoot. The Indian strain is also more frequently resistant to antifungal drugs, which has been attributed to the overuse of common antifungals by the public. Consequently, the disease is hard to control, and occurrence of the dermatophyte is on the rise [35,56].

A case of host shift has been noted earlier in the dermatophyte *Trichophyton equinum* infecting horses. Kandemir et al. [57] describes *Trichophyton tonsurans*, which causes tinea capitis in humans as the anthropophilic counterpart of *T. equinum*. Here, rapid evolution towards anthropophily may have occurred by the clonal emergence of mating types.

### 5.2. Sporotrichosis

In recent years, zoonotic sporotrichosis has become an emerging public health issue in Latin America and has taken the proportion of an epidemic [58]. In the state of Rio de Janeiro alone, cases have escalated from a few hundred in the late 1990s to more than 10,000 in the year 2017 [59]. Two species are the cause of the ongoing feline epizootic and epidemic: *Sporothrix brasiliensis* and *Sporothrix sckenckii* sensu stricto, the former predominating [59].

The emergence of *Sporothrix brasiliensis* in cats in Brazil has been linked to a lifestyle shift of a saprobic *Sporothrix* species. The acquired ability of the fungus to survive at the relatively high body temperature of cats may be related to global warming [58,60]. The fungus easily spreads among cats through scratches and bites in fights. Cases in humans occur exclusively by zoonotic transmission. An epidemiological relationship has been observed between the number of infected cats in the urban areas of Brazil and diagnosis in humans [61]. Although cats are the primary vector, *Sporothrix brasiliensis* also prevails among dogs and rats [62]. As for dermatophytes, zoophilic *Sporothrix brasiliensis* is more virulent than previously known species. Alarmingly, the fungus is also insensitive to some antifungal treatments [62,63]. The prevalence of the disease keeps increasing in Brazil and is now spreading to adjacent Argentinian states [61].

As opposed to the Brazilian species, *Sporothrix sckenckii* sensu stricto occurs worldwide. Zoonotic transmission has been reported mainly from cats, but also dogs, squirrels, rats and armadillos. *Sporothrix globosa*, *S*. *mexicana* and *S*. *luriei*, also occur in humans but are commonly acquired through the inhalation of conidia rather than animal transmission [62,64,65].

### 5.3. Histoplasmosis

Approximately 500,000 people suffer from histoplasmosis annually, 100,000 develop disseminated histoplasmosis and 25,000 die from the disease [1]. Histoplasmosis is emerging worldwide. Originally, the disease was endemic in Ohio, Mississippi; St. Lawrence River Valley in America; and in sub-Saharan Africa [66,67], but it now extends to the Caribbean, Southeastern Asia and South and Central America [68]. As the disease is often misdiagnosed (e.g., as tuberculosis or emergomycosis) and notification of infection to the authorities is not mandatory in many countries, its geographical prevalence and distribution may still be underestimated [69,70].

The causative agent of the disease is the dimorphic fungus *Histoplasma capsulatum*, which was first isolated in Mexico. Recent studies have revealed that *H. capsulatum* is a species complex rather than a single species. Sepúlveda et al. [71] established a number of sibling species within the *H. capsulatum* complex on the basis of population genomic data, each with an approximately restricted geographic distribution. The genetic diversity between populations is considerable [72,73], and the fungus comprises at least four distinct genetic groups of differing virulence [71].

Histoplasmosis, once known as the “cave sickness” [74], has been strongly associated with bats. Several species and populations of Chiroptera, belonging to Molossidae, Phyllostomidae, Vespertilionidae and Emballonuridae, have been reported with the disease [75,76]. Histoplasmosis has also been reported in birds, but only sporadically [73]. However, rather than being zoonotic, the causative agent, *H. capsulatum,* thrives in the feces of bats and birds and in soils enriched with their fecal matter [25,66]. Interestingly, an overlap was found between the geographical occurrence of infected bats and regions of histoplasmosis endemicity [77]. As infected bats travel for long distances and migrate, they spread the pathogen [70,78]. Among mammals, bats are the second most speciose group after rodents; they have a worldwide distribution and live in colonies, which are sometimes found in close proximity to humans. These features make bats a continued source of emerging pathogens [36]. However, encroachment into wildlife habitats related to touristic activities such as bird and bat watching, caving or construction and excavation work should be regarded as the main reasons for the spillover of histoplasmosis in humans [79,80].

A wide range of mammalian hosts including bears, monkeys, apes and several felines have also been reported with *H. capsulatum* infection [81]. Teixeira et al. [73] found that isolates of *H. capsulatum* from specific mammalian hosts formed monophyletic clades, suggesting that mammals play a key role in the diversification (e.g., strains) and the mechanism by which the fungus disperses. Other authors have also discussed the significance of animal hosts in the evolution of new pathogenic fungal species [82]. Wide host range is yet another characteristic of an emerging disease [83].

Histoplasmosis is typically contracted by the inhalation of airborne conidia from the environment and usually affects AIDS patients. However, cases have recently been reported in otherwise immunocompetent individuals [68,84,85]. In general, histoplasmosis symptomatology ranges from asymptomatic in immunocompetent patients to fatal in immunocompromised ones [86]. In addition, the severity of the infection also depends on the inoculum size and age of the individual [80], but unexpected cases do occur. In a recent outbreak, members of a film crew suffered from histoplasmosis after working in a vampire bat cave. One of them, a young man in his thirties with no underlying conditions, suffered from acute pulmonary histoplasmosis, as he occasionally removed his mask [87].

### 5.4. Cryptococcosis

Beyond the tropics and the sub-tropics, cryptococcosis is now recognized as one of the deadliest fungal diseases worldwide [8,88]. Since the first case report in the mid 1980s, the epidemiology of cryptococcosis has evolved [89]. The emerging invasive fungal disease now kills both treated and untreated patients with and without underlying immune defects [89], causing a death toll of over 180,000 annually [20,90]. The course of the disease can be insidious or can cause a range of symptoms. Meningoencephalitis is common in HIV-positive and other immunosuppressed patients and is often caused by *Cryptococcus neoformans*. Concurrently, *C. gattii* is increasingly being diagnosed in immunocompetent individuals causing pulmonary infection [91].

In terms of diseases related to and dispersed by animals, *Cryptococcus neoformans* is of main concern as it typically thrives in bird excreta. The habitat of *Cryptococcus gatti* comprises of plants, particularly eucalyptus [91]. However, species belonging to both the *C. neoformans* and *C. gattii* species complexes shift to an infective yeast form once they go through the respiratory system of mammals. Numerous cases, outbreaks and epidemics of cryptococcosis by the two species complexes have been documented in farmed and companion animals: birds, cattle, horses, cats and dogs, among others [92,93,94]. The risk of human cryptococcosis by these infected animals should not be neglected given that they are part of our daily life and have a high potential to contaminate the environment.

Insightful discoveries and advances have been made in the molecular characterization, diagnosis and treatment of cryptococcosis [89,95,96,97]. However, the scarcity of antifungal treatments to treat mycosis and the emergence of antifungal resistance challenges the goal to curb mortality rates [96].

### 5.5. Emergomycosis

Emergomycosis, previously known as adiaspiromycosis for the giant cell variant, is caused by members of *Emergomyces* [98]. This genus is particularly interesting due to the numerous drastic changes that have been made to its classification within just a few years [99]. The previous type species *Emmonsia parva* has been moved to *Blastomyces*. Additionally, *Emmonsia crescens* and *E. soli* have been reassigned to *Emergomyces,* although the morphology and size of their invasive stage (a determinant characteristic for delineating dimorphic fungi at a generic level) differ significantly from other members of the genus. Additionally, *Emergomyces* species show considerable variation in their virulence and clinical manifestations [99,100,101]. Previously, due to the unconventional characteristics of *Emergomyces*, the identification of species causing emergomycosis was difficult and ambiguous. These were collectively referred to as emmonsia-like species (*Emmonsia* sp.) in reference to the resemblance in symptoms of the disease (adiaspiromycosis) caused by *Emmonsia crescens* and *Emmonsia parva* [99,100]. Recent phylogenetic analysis separated them into five distinct species belonging to *Emergomyces* (*E. africanus, E. canadensis, E. europaeus, E. orientalis* and *E. pasteurianus*).

Currently, seven species are known to cause emergomycosis. These include exclusive human pathogens as well as pathogens common to both humans and other animals. *Emergomyces crescens* is normally associated with small burrowing mammals, particularly Cricetidae and Muridae rodents [48,102]. Cases in other rodents and humans occur occasionally [103]. The disease has thus been considered potentially zoonotic with small rodents and their predators as vectors; however, conclusive evidence is yet to be found. Jiang et al. [48,99] suggested that zoonotic infection by *E. crescens* remains highly unlikely, as a higher frequency of infection by the fungus appears in hosts with body temperatures relatively lower (35.0–37.5 °C) than that of humans. *Emergomyces africanus* with increased prevalence, causing hundreds of deaths in HIV patients yearly, is exclusively found in humans. Yet emergomycosis remains of particular interest when dealing with animal-associated fungal diseases in humans, as it strongly resembles histoplasmosis in its clinical presentations and diagnostic test results. Anthropophilic *Emergomyces* species, such as *E. africanus*, and *Histoplasma capsulatum* proliferate by forming thin-walled, budding yeast and yeast-like cells, leading to disseminated infection. Dissemination commonly involves the spleen, liver, gastrointestinal tract and bone marrow [104,105]. When the infective agent is sampled and cultured, swollen conidiophores and florets of conidia form fluffy whitish-yellow mycelium in both anthropophilic *Emergomyces* and *Histoplasma capsulatum* [99]. However, the mycelial form varies considerably, and if successfully cultured, it can be used as a distinguishing characteristic [44,99]. Ultimately, molecular methods provide a more accurate means for identification [39,106].

### 5.6. Talaromycosis

*Talaromyces marneffei* (previously known as *Penicillium marneffei*) infects around 50,000 HIV positive patients every year in endemic regions [18]. However, in the last decade, a change in the epidemiology of *Talaromyces marneffei* has been observed. The fungus once considered exclusively AIDS-related now shows association with other types of impairment of cellular immunity such as disorders related to mutation of the STAT-1 gene [107,108]. In Southern China and East India, immunocompetent individuals also seem to be involved [85,109,110,111].

Talaromycosis is described as a soft-tissue infection, but recently, Li et al. [112] stated the radiological findings of bone involvement in non-HIV talaromycosis patients, mainly osteolytic bone destruction in flat bones. Concerns arise as the clinical manifestations of non-HIV talaromycosis develop rapidly, are non-specific and occur concurrently with other opportunistic fungal infections, often leading to misdiagnosis [110,112]. Surprisingly, disease mortality was found to be higher in non-HIV patients than in HIV-positive patients [113].

A newly reported case in a patient who had no recent contact with any known source or reservoir of infection [110] has brought back uncertainties around the ecology and transmission of *Talaromyces marneffei*. The fungus comprises the sole dimorphic species of *Talaromyces* [114]. Little is known about the causative agent of talaromycosis. Its natural environmental niche is still debated, and the mechanism through which it invades the host is poorly understood [115]. Infection is presumed to occur through inhalation of conidia from the environment [116]. Zoonotic transmission has also been speculated as strains infecting bamboo rats and humans are genetically similar [117]. A case of disseminated talaromycosis has also been reported in a male who had consumed bamboo rat meat [109]. However, further evidence is needed to confirm the hypothesis of direct transmission from rodents to humans. Since the first isolation of the fungus from the liver of bamboo rats, subsequent studies uncovered the natural occurrence of the fungus in plant materials and soil associated and non-associated with the rodent [14,15,16,17,115].

## 6. Conclusions

Fungal diseases typically associated with animals continue to emerge in humans mainly due to human modern activities. With increased movement and development along with environmental changes, emerging fungal infections are on the rise and require close surveillance. The emergence of animal-associated diseases in humans is noted as a result of either an increase in the number of cases or as host switch of a known species. In all cases, assessing the risk that these fungi pose to public health may be challenging, as it requires interdisciplinary collaboration. Infections of humans and domestic animals have been studied thoroughly, but knowledge on wildlife habitats is fragmented. Tackling this gap requires collaborative research among many fields, including taxonomy, ecology and epidemiology. The basis of understanding and forecasting epidemics is to trace the source of the disease. Wildlife should be given close attention, yet it should not be spuriously considered as the source of all threatening diseases. If emerging diseases can be predicted early, then preventive methods (e.g., novel pharmaceutical agents) or other methods can be developed in a timely manner [1,3,7]. Though this review is centered on humans and public health, successfully tackling emerging fungal diseases will require a One Health approach, whereby humans, other animals and the environment are considered.

## Data Availability

Not applicable.
